# Central adrenal insufficiency and diabetes insipidus as potential endocrine manifestations of COVID-19 infection: a case report

**DOI:** 10.11604/pamj.2021.38.222.28243

**Published:** 2021-02-26

**Authors:** Abu Baker Sheikh, Muhammad Ali Javaid, Abdul Ahad Ehsan Sheikh, Rahul Shekhar

**Affiliations:** 1 Department of Internal Medicine, University of New Mexico Health Sciences Center, Albuquerque, New Mexico (NM), USA,; 2 Department of Internal Medicine, Dow Medical College, Karachi, Pakistan,; 3 Department of Internal Medicine, The Wright Center for Graduate Medical Education, Scranton, Pennsylvania (PA), USA,; 4 Department of Internal Medicine, Division of Hospital Medicine, University of New Mexico School of Medicine, Albuquerque, New Mexico (NM), USA

**Keywords:** COVID-19, central adrenal insufficiency, central diabetes insipidus, case report

## Abstract

SARS-CoV-2 infection, responsible for the coronavirus disease-2019 (COVID-19) has rapidly spread, causing a global pandemic. COVID-19 can affect any organ system in the body due to overwhelming dysregulated immune response and long-term effects of the disease is still unknown. Endocrine complications associated with COVID-19 is exceedingly rare. Here we present a unique case of a 44-year-old female who developed adrenal insufficiency and central diabetes insipidus following COVID-19 infection.

## Introduction

Coronavirus disease (COVID-19) has become a leading global pandemic health calamity affecting millions around the world. Although initially thought COVID-19 as primarily a disease of the respiratory system, it has multiple extrapulmonary manifestations [1]. Due to dysregulated immune response, it can affect any organ system in our body including cardiovascular, neurological, renal, gastrointestinal systems, and in rare instances endocrine system causing numerous complications [1]. We present a novel case of a 44-year-old female with COVID-19 pneumonia who later on her disease course developed adrenal insufficiency and central diabetes insipidus.

## Patient and observation

Forty-four-year-old female patient with a past medical history of type 2 diabetes mellitus diagnosed three years ago treated with insulin glargine and dulaglutide presented with subjective fevers and progressively worsening shortness of breath. She was diagnosed with COVID-19 infection with nasopharyngeal reverse transcriptase polymerase reaction (RT-PCR) at an outpatient clinic 12 days ago after presenting with chills, myalgias, loss of taste, fatigue, and malaise.

**Figure 1:**
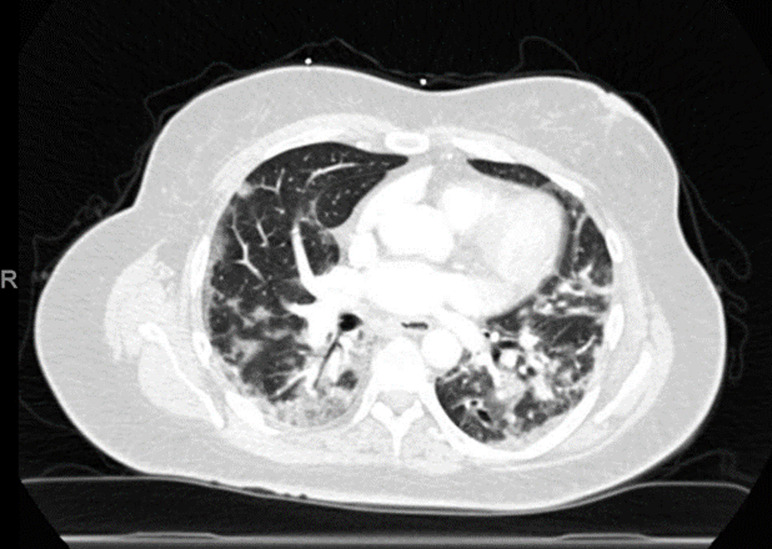
chest computed tomography of patient with COVID-19 pneumonia showing bilateral multifocal opacities in lungs

On admission, her temperature was 37.8°C, a pulse of 75 beats per minute, blood pressure 112/72mmHg, mild tachypnea with a respiratory rate of 28 breaths per minute with oxygen saturation of 84% on room air and was immediately put on four liters of supplemental oxygen via nasal cannula. Physical exam was significant for bilateral coarse breath sounds left worse than right. Repeat COVID-19 RT-PCR test was positive. Chest imaging and computed tomography chest angiogram reveal multifocal pneumonia (Figure 1). She was started on dexamethasone 6 milligrams (mg) for 10 days and remdesivir for 5 days. Her laboratory values are provided in Table 1.

**Table 1:** laboratory work

Lab variable	On admission	Day 13 of hospitalization	On discharge	Reference values
White Blood Cells / mm^3^/ul	11.2	12.7	9.7	4.5-11
Hemoglobin / g/dl	11	13.3	12	13.5-17.7
Platelets / per mm^3^/ul	338	420	203	150-450
Serum sodium / mmol/L	136	138	141	134-144
Serum potassium / mmol/L	3.6	3.4	3.7	3.5-5.1
Serum creatinine / mg/dl	0.94	0.81	0.63	0.6-1.7
TSH / IU/ml	1.83	N/A	N/A	0.36-3.7
Free T4 / pg/ml	0.8	N/A	N/A	0.7-1.6
AST / Unit/L	19	N/A	22	5.0-40
ALT / Unit/L	29	N/A	33	14-67
ALP / Unit/L	62	N/A	74	38-150
Total Bilirubin / mg/dL	0.8	N/A	1.1	0.3-1.2
Albumin / g/dL	2.2	N/A	3.1	3.4-4.7
Direct Bilirubin / mg/dL	0.3	N/A	0.8	0.1-0.4
Troponin / ng/ml	<0.017	<0.017	<0.017	0.06
Brain Natriuretic Peptide / pg/ml	149	169	N/A	0-125
COVID-19 PT-PCR NP	positive	N/A	negative	
Random cortisol mcg/dl	N/A	1.1	N/A	8.0-25
Urine output Liter/day	2.2	6.2	1.7	8.0-2
Urine osmolality mOsm/kg	N/A	302	N/A	500-850
Urine sodium mEq/day	N/A	107	N/A	40-220
Hemoglobin A1c%	9.8	N/A	N/A	<5.7
ACTH pg/ml	N/A	56	N/A	10-60

On day 12 of hospitalization, she started to complain of bouts of dizziness with nausea. She was found to be hypotensive with systolic blood pressures in the high 70s to low 80s mmHg. She received two liters of ringer lactate boluses with no change in blood pressure readings. She was also found to have increased urine output of more than 6 liters per day. Further investigation revealed random cortisol level of 1.1ug/dl, sodium of 139mg/dl and potassium of 3.4mg/dl, urine osmolarity of 302 mOsm/kg, urine sodium of 107mEq/day, and serum osmolarity of 298mOsm/kg. Adrenocorticotropic hormone (ACTH) level of 56 pg/ml. The patient was suspected of central adrenal insufficiency and diabetes insipidus. ACTH stimulation test exhibited an appropriate increase in cortisol levels, cortisol levels were found to be 2.2, 10.8 and 20.4 at 0, 30 and 60 minutes respectively. She was started on desmopressin 0.1mg for concern for diabetes insipidus after which her urine osmolality increased to 764mOsm/kg and urine sodium increased to 153mEq/day and her urine output significantly decreased to 3.2 liters per day. She had no history of head trauma, exogenous steroid use, vision problems, heat or cold intolerance, headaches, smoking, or pregnancy complications.

The patient was initially administered 25mg of hydrocortisone twice daily which was titrated down to 25mg in the morning and 10mg in the evening per endocrinology team recommendation. Magnetic resonance imaging (MRI) of the pituitary gland was negative for any pituitary mass, acute hemorrhage, or malignancy. She continued to receive hydrocortisone and her blood pressures improved and her symptomatology resolved. She was not requiring oxygen and was discharged with hydrocortisone dose to 15mg in the morning and 5mg in the evening with instructions to increase her dose to 25mg/10mg if she became symptomatic. She was also prescribed desmopressin 0.1mg daily for her diabetes insipidus. An appointment was set up with Endocrinology and Primary care physician, unfortunately, she lost to follow up after the discharge.

## Discussion

COVID-19 has been known to cause complications of pre-existing medical conditions such as diabetes mellitus and adrenal insufficiency [1]. However, recent studies have also demonstrated it to be a precipitating factor for the emergence of endocrine disorders in previously healthy patients including irregularities of thyroid function as well as dysfunction of the hypothalamic-pituitary-testicular axis was also noted owing to immune-mediated damage [2, 3].

Previously central adrenal insufficiency has been reported in the patients infected with SARS-CoV. It is implied that the hypothalamic-pituitary-adrenal axis impairment is probably a delayed pathological complication post-SARS rather than merely be ascribed to suppression by exogenous steroid use. Leow *et al*. had proposed the possibility of a reversible hypophysitis or direct hypothalamic damage that could have led to a state of hypothalamic-pituitary dysfunction [4]. Other mechanisms suggested include the production of certain amino acids by SARS mimicking ACTH which results in host antibody production resulting in adrenal insufficiency [5]. Furthermore, the release of inflammatory markers such as Interleukin-1, Interleukin-6, and Tumor necrosis factor-α is thought to decrease ACTH production by the pituitary gland [6]. The damage was reversible in cases of SARS-COV, however, with COVID-19 the question of the damage currently remains undetermined. Central diabetes insipidus can have multiple underlying etiologies such as idiopathic, germ cell tumors of central nervous system, empty sella syndrome, and pituitary adenoma [7]. Both pituitary gland and hypothalamus express ACE 2 receptors which are a potential target of SARS-CoV-2 [8]. Considering the high frequency of neurological symptoms, one can assume that SARS-CoV-2 may affect the hypothalamus-pituitary as well, directly or via immune-mediated hypophysitis [9].

Heidarpour *et al*. reported a suspected case of adrenal insufficiency in a 69-year-old man with severe COVID-19 infection leading to vasopressor resistant hypotension, his random cortisol level was 12μg/dl. It was managed with intravenous hydrocortisone in the critical care setting and was discharged after a prolonged hospitalization. No ACTH stimulation test or pituitary imaging was performed on this patient, he responded well to hydrocortisone therapy [10]. We here to our knowledge, report a first case of central adrenal insufficiency confirmed with ACTH stimulation test and diabetes insipidus as a sequela of COVID-19 infection with a good response to oral hydrocortisone and desmopressin therapy.

## Conclusion

We recommend physicians should have high clinical suspicion for endocrine disorders in any patient with confirmed COVID-19 infection who presents with electrolyte derangements, nausea, vomiting with or hypotension and adrenal insufficiency should be ruled out in such patients by appropriate workup. Prompt diagnosis and treatment can result in decreased morbidity and mortality among such patients. More studies and long-term follow-up of recovered COVID-19 patients is recommended to recognize endocrine post-COVID-19 complications, clinical course, and recovery.
